# Accessing the Influence of Community Experience on Brand Loyalty Toward Virtual Brand Community: Developing Country Perspective

**DOI:** 10.3389/fpsyg.2022.865646

**Published:** 2022-04-26

**Authors:** Zhounan Huangfu, Yaohui Ruan, Jing Zhao, Qiaqia Wang, Lei Zhou

**Affiliations:** ^1^Design College, Zhoukou Normal University, Zhoukou, China; ^2^Valaya Alongkorn Rajabhat University, Khlong Luang, Thailand; ^3^Faculty of Fine Arts, Srinakharinwirot University, Bangkok, Thailand; ^4^Academy of Fine Arts, Zhengzhou Normal University, Zhengzhou, China; ^5^Art Design College, Henan University of Engineering, Zhengzhou, China

**Keywords:** virtual brand community, community experience, brand community identity, brand loyalty, social media marketing

## Abstract

With the development of information technology, more and more companies have taken the initiative to build virtual brand communities to strengthen the connection between brands and customers and create brand loyalty, but existing research lacks a clear explanation of how the customer community experience of virtual brand communities affects brand loyalty. In response to this question, this paper investigates the role and mechanism of different types of community experiences, namely information experience, entertainment experience and interactive experience, generated by customers’ participation in community activities on brand loyalty based on the customer experience perspective. The findings show that different types of community experiences have a significant impact on brand loyalty, and which community identity is partially mediated in all of them. Further analysis for segmented populations in virtual communities revealed significant differences in the above mechanisms for members of different genders. The findings of this paper are important guidance for companies to manage virtual brand communities and create brand loyalty.

## Introduction

With the accelerated development of new-generation information technology, the connection between enterprises and consumers has migrated from real to virtual platforms, and virtual brand communities based on virtual platforms have emerged and attracted sufficient academic attention (Hakala et al., 2017; Stoycheff et al., 2017; Wang et al., 2021). A virtual brand community is an online community based on social relationships and social connections among consumers of a brand that is not limited by geographic scope (Ul Islam and Rahman, 2017; Kaur et al., 2020; Rubio et al., 2020). The emergence of virtual brand communities has changed the way companies communicate with consumers, and more and more companies are trying to use virtual communities to strengthen their connection with consumers (Li, 2021). Due to the tremendous increase in consumer autonomy in virtual communities, consumers are no longer passive recipients of information, but active content creators. This means that companies can no longer forcefully push information to consumers, but rather strive to provide a good experience for consumers in the hope that consumers’ experiences in virtual brand communities will translate into consumer brand loyalty (Coelho et al., 2018). Research has shown that traditional brand communities are mainly formed spontaneously by consumers, however, with the development of social media, companies are increasingly aware of virtual brand communities as an important tool to strengthen the connection between brands and consumers (Yang et al., 2021), and more and more companies are taking the initiative to establish virtual brand communities (Sylvia Christianti et al., 2021; Yang et al., 2022). The important goal of virtual brand communities is to create a platform for a series of consumer activities that strengthen the connection between brands and consumers and create brand loyalty among consumers (Wang et al., 2021).

Existing research shows that the positive role of virtual brand communities has attracted widespread academic attention, and a series of discussions have been conducted to obtain more consensus, mainly in the following aspects. First, although research has identified community experience as a key factor influencing the marketing of virtual brand communities, its role is usually examined as a holistic concept (Sha et al., 2010). Community experience facilitates consumers to form good relationships with companies, brands, products, and other consumers (Ball and Barnes, 2017; Wang et al., 2019). While these findings can reveal the positive aspects of community experiences, they struggle to reveal the differential impact of different dimensions of the community experience generated by community activities, limiting the usability of the study’s findings as they do not distinguish how the different components of this community experience affect consumers’ brand loyalty. Secondly, the existing literature generally agrees that consumer community identification of virtual brand communities is an important measure affecting their performance (Xu and Li, 2014), but previous studies tend to focus on the impact of the degree of consumer participation in virtual brand communities on community identification or product attitude aspects, and as a result, the mechanism of the effect of consumer community experience on brand loyalty is ignored, However, previous studies have tended to focus on the effect of the level of consumer participation in virtual brand communities on community identification or product attitudes, and as a result, the mechanism of the effect of consumer community experience on brand loyalty has been overlooked, while actually creating brand loyalty is the value of establishing virtual brand communities for companies (Krisvianti and Triastuti, 2020; Wang et al., 2021). Therefore, previous studies lacked research on the antecedents that lead to the creation of community identity and its impact results on brand loyalty. Third, although researchers have confirmed that consumers’ community experiences in virtual brand communities can contribute to the formation of positive attitudes and behaviors (e.g., brand loyalty) toward that community, company, and product, the intermediate processes by which community experiences influence brand loyalty relationships have rarely been explored (Nambisan and Watt, 2011; Pillay and Mpinganjira, 2019). Indeed there is a general inconsistency between consumers’ attitudes and behaviors. As pointed out in the study of Bhattacharjee et al. (2021) which: existing research often lacks an exploration of the mediating relationship between virtual brand community experience and brand loyalty, which leads to a lack of clear explanation of the mechanism of virtual brand community effect.

Apparently, the existing studies have been discussed in a series and have gained more consensus. However, the formation of consumer community identity in a virtual brand community engagement context does not occur naturally, but is formed gradually during the consumer engagement process (Kaur et al., 2020). This implies that it has become an important topic to explore the impact of consumers’ community activities on virtual brand communities from the perspective of consumers’ community experiences (Goncalves et al., 2021). To this end, this study takes a consumer experience perspective, specifically distinguishing the different component facets of community experience, namely information experience, entertainment experience, and interactive experience, and examining the impact of all three on brand loyalty and the mediating role of community identity in it. Specifically, this study takes “OPPO community,” a virtual brand community established by the company, as the research object, and sets community experience as the antecedent influencing factor, community experience as the mediating variable, and brand loyalty as the outcome variable, and investigates the mechanisms of information experience, entertainment experience and interactive experience on brand loyalty in virtual brand community experience through structural equation modeling. The study examines the mechanisms of community experience, entertainment experience and interactive experience on brand loyalty through structural equation modeling, and examines the significant differences in the effects and mechanisms of community experience, entertainment experience and interactive experience on brand loyalty, so as to provide new strategies for the business practice of virtual brand communities.

## Theoretical Foundation and Hypothesis Development

### Literature Review

#### Social Identity Theory

Social identity theory suggests that people tend to categorize themselves and others into different social groups, identify with their own group through categorization, and consequently develop ingroup preferences and outgroup biases (Tajfel, 1982; Ashforth and Mael, 1989). Often, the self is included in a category during categorization, and characteristics that fit the in-group are assigned to the self (Turner, 1985). Under the influence of pro-social thinking, as one of the main ways to build brand loyalty to satisfy consumers’ self-definition, companies can make it easier for consumers to distinguish companies from their competitors through consumers’ participation in virtual brand community experiences, and use these community experiences of consumers as a basis for positioning and differentiating themselves in the social environment, and through social comparison and positive differentiation, individual identities around these community experiences Through social comparisons and positive distinctions, the contrast between individual identities around these community experiences and social identities around salient group categories gives rise to consumer self-concept definitions based on community experiences, i.e., social identity, and when comparing between groups, individuals tend to exaggerate the differences between them on specific traits and give more positive evaluations to ingroup members, resulting in biased evaluations and behaviors in favor of the group to which they belong (Schau et al., 2009; Lin and Xie, 2018).

Community identity is a special form of expression of social identity, and the prerequisite for individuals to participate in interaction, communication and shopping is to establish a virtual identity for themselves, which plays a key role in interpersonal communication in a technologically mediated environment (Liao et al., 2019). It reflects the virtual self personal that the user wants to express and present to others. In virtual brand communities, users can build and express themselves using freely scalable symbolic tools (short videos, personalized signatures, user avatars), etc., and create one or even multiple online “virtual identities” to showcase multiple aspects of their selves (Wu and Liu, 2018). As in the real world, users have the dual motives of satisfying their sense of belonging and preserving their individuality in the process of participating in virtual brand communities, i.e., the construction of virtual identities reflects the need of users for community identity in the real world, by finding the difference between “I” and “we” and by finding the difference between “we” and “them” to gain community identity. To gain self-identity by finding the difference between “me” and “us,” and to gain community identity by finding the difference between “us” and “them” (Yang and Yin, 2019).

#### Community Experience

Experience is a unique emotion developed by consumers in a specific context, with the help of use, interaction and creative behavior, in direct or indirect contact with a company. Experience enables consumers to represent themselves through unique emotions, and consumers pursue experience for the direct purpose of seeking a unique emotional need (Li, 2019). Relevant studies have shown that experience is a higher level of spiritual needs of individuals, a holistic feeling obtained by individuals who are fully engaged in activities and realize their self-worth, a feeling that is usually difficult to express in words. Different scholars have put forward different views and ideas, including sensory experience, immersion experience, flow experience, peak experience, transcendental experience, and smooth experience (Maslow, 1987; Arnould and Price, 1993; Vitterso et al., 2000). There are also studies that consider experience as a subjective feeling formed during the interaction between companies and consumers. The main studies include Holbrook’s classification of experience as experiential, entertainment, performance mania and didactic Holbrook (2001) and Gentile et al. (2007) classification of experience as sensory, emotional, cognitive, practical, lifestyle and relational.

With the development of information technology and the rise of virtual platforms, consumer experience in virtual brand communities has received attention from scholars (Hsu and Lu, 2004; Mathwick and Rigdon, 2004; Brakus et al., 2009), and the multidimensional concept and structure of community experience has been studied. Among the representative scholars are Sha et al. (2010) and Nambisan and Watt (2011). Nambisan and Watt (2011) defined community experience as “the full range of experiences that consumers have during virtual community interactions” based on the virtual context of the Internet. Sha et al. (2010) classified community experience into three dimensions: information experience, entertainment experience and interaction experience based on the virtual brand community context. In addition, some other scholars have also studied the dimensions of community experience. In general, scholars generally agree that virtual community experience includes three dimensions: information experience, entertainment experience, and interaction experience. Therefore, combined with the virtual brand community context, this study draws on Sha et al. (2010) and Nambisan and Watt (2011) and agrees that community experience mainly reflects the feelings that consumers get from different activities in the virtual brand community, and thus will classify its dimensions into information experience, entertainment experience and interactive experience.

### Hypothesis Development

#### Community Experience and Brand Loyalty

Given the important role of brand loyalty in corporate performance, exploring the factors that promote brand loyalty such as experience and brand trust has become a hot topic in academia (Chaudhuri and Holbrook, 2001). With the emergence and development of virtual communities, the social scope of consumers has gradually expanded from the traditional physical environment to the virtual. Today, companies are gradually realizing that virtual brand communities can help them manage customer relationships, enhance their brand influence, and strengthen consumer brand loyalty. Undoubtedly, virtual brand communities have become an effective way and strategy for companies to build consumer brand loyalty (Zhou and Zheng, 2011). Scholars have long noted the existence of some relationships between consumer experience and brands. For example, Park et al. (1986) have pointed out that brand value consists of functional, experiential, and symbolic values, and that the intrinsic experiential value of a brand is an important consumer need. Morgan and Hunt (1994) and Wu et al. (2009) also argue that brand relationships are formed by a sequence of consumer experiences and exposure to a brand (Morgan and Hunt, 1994; Wu et al., 2009). However, they did not investigate the role and mechanism of community experience on brand loyalty in the context of social media. Based on this, it can be found that in the virtual brand community context, information experience, entertainment experience and interactive experience are the important elements that constitute the virtual brand community. In other words, the brand virtual community is a three-dimensional concept consisting of information experience, entertainment experience and interactive experience (Sha et al., 2010; Nambisan and Watt, 2011). Therefore, in virtual brand communities, consumer community experiences (information experiences, entertainment experiences, and interactive experiences) drive community identity in three main ways.

The first aspect, information experience, refers to the experience of consumers who feel the usefulness of the information itself when they access or share relevant content on virtual platforms (Sha et al., 2010; Nambisan and Watt, 2011). In the age of social media, consumers have changed from passively receiving information from companies to relying on the attitudes, opinions and behaviors of other consumers about products and seeking solutions to technical problems with products. Since virtual brand communities are virtual platforms built around a brand, they can effectively gather a large amount of information about the brand, and consumers’ information experience in the community makes them lower the perceived risk of purchasing the product and the threshold of choosing again (Wu et al., 2009; Wang et al., 2019). Based on this, it can be inferred that in the virtual brand community context, consumers’ information experience contributes to the formation of consumers’ community identity.

The second aspect, entertainment experience, expresses the pleasant or relaxing feeling that consumers get by participating in the virtual platform community (Sha et al., 2010; Nambisan and Watt, 2011). Seeking entertainment has become an important motivation for consumers to participate in social media today, and some studies have shown that companies regularly generate entertaining content to gain consumers’ attention and favorability (Li, 2012, 2021). Based on this, it can be inferred that in the virtual brand community context, consumers’ entertainment experience contributes to the formation of consumer community identity.

The third aspect, interactive experience, refers to the experience that consumers gain by communicating and interacting with other members in the virtual platform community (Sha et al., 2010; Nambisan and Watt, 2011). Virtual communities are inherently interactive in order to persist. In social interactions, consumers can find members with similar interests, establish closer relationships with other consumers, and develop social status and a sense of belonging in the community (Wang et al., 2019). Based on this, it can be inferred that in the context of virtual brand communities, consumers’ interactive experiences contribute to the formation of consumer community identity. As such, we expect that community Experience will drive consumers to attain higher community identity.

**Hypothesis 1:** Information experience is positively associated with consumers’ community identity.
**Hypothesis 2:** Entertainment experience is positively associated with consumers’ community identity.
**Hypothesis 3:** Interactive experience is positively associated with consumers’ community identity.

#### Community Identity and Brand Loyalty

Research shows that the relationship between consumers and brands is not simply between consumers and brands, but is a dynamic relationship between “consumers-brands-other consumers” because consumers are not isolated consumers of products or brands; individuals are often linked by their status as consumers of a brand, sharing The meaning of the product, the value of maintaining the brand (Muniz and O’Guinn, 2001; Laroche et al., 2012; Kaur et al., 2020). Brand communities are an important vehicle for the “consumer-brand-other-consumer” relationship, as evidenced by the rapid development of social media and information technology, where more and more consumers are participating in virtual brand communities, sharing brand knowledge and brand stories, and meeting other consumers who use the same brands. This is reflected in the rapid development of social media and information technology, where more and more consumers are participating in virtual brand communities, sharing brand knowledge and stories, and meeting other consumers who use the same brands. In this process, the information, entertainment, and interaction that consumers receive in the community directly affect the relationship between consumers and the brand community, and the closeness of the relationship between consumers and the community further affects consumers’ perception of the brand (Weijo et al., 2019).

It has been shown that in virtual brand communities, consumers’ community identification is an important measure of the emotional connection between consumers and the virtual brand community, and an important antecedent influence on consumers’ continued engagement in the community (Kaur et al., 2020). Community identity in a virtual brand community is the extent to which members see themselves as members of that brand community (Algesheimer et al., 2005). Recent research has further shown that consumers who join virtual communities because of shared brand preferences also identify with the virtual community during community interactions (Sicilia and Palazón, 2008). Consumers’ shared values and experiences in a virtual community are the basis for the creation of a community identity. The identity of a virtual brand community provides a social status for consumers and fosters a sense of belonging to the community. As consumers develop a sense of belonging in a virtual brand community, this sense of belonging is strengthened through interaction with other consumers, which leads to continued participation in the virtual community, which in turn pulls consumers to deepen their knowledge and love of the brand and ultimately promotes the formation of brand loyalty.

Some studies have shown that virtual brand community identity will increase consumers’ favorable perception of community platform companies and make them more willing to share their knowledge with the company (Qin, 2020; Bhattacharjee et al., 2021). It has also been shown that consumers’ identification with virtual brand communities can directly contribute to brand identity, while at the same time promoting consumers’ commitment to the community, which in turn positively influences consumers’ brand commitment (Wongsansukcharoen, 2022). This suggests that consumers are likely to project their feelings toward the brand community through a process of emotional migration, resulting in brand loyalty (Bhattacharjee et al., 2021). Finally, by participating in the community, the brand’s products act as a medium of communication and exchange among the community members and are an expression of the members’ community identity, and consumers are more likely to purchase products belonging to the brand to reinforce their community identity (Li, 2012, 2021). Based on the aforementioned arguments, we hypothesize the following:

**Hypothesis 4:** Community identity is positively associated with consumers’ brand loyalty.

#### Mediating Role of Community Identity

Existing research suggests that consumers’ emotional relationships with brands in virtual brand community usage contexts need to undergo a gradual process of experience (Wu et al., 2009). Therefore, as individuals’ experience in virtual brand communities deepens, consumers’ identification with the community is strengthened, and eventually they develop an emotional bond to the brand because of their experience in the community, and then move away from the previous brand choice based on economic benefits and transform from community identification to brand loyalty (Bhattacharjee et al., 2021).

Research suggests that information experiences may enhance consumers’ community identity. Individuals need two basic elements to form a community identity: one is to have the identity of a specific group, and the other is that individuals are aware of the value and emotional significance of this identity (He and Yan, 2018). Clearly, forming a community identity requires not only that community members are aware of their identity as community members, but also that they perceive the value and meaning that comes with being a community member, otherwise they cannot form an identity with the community. Since information experience enables consumers to obtain useful information about product use and purchase in community browsing content, eliminating uncertainty about product use and other aspects (Zhu et al., 2016), this makes consumers directly deepen their understanding and love for the brand on the one hand, and makes community members perceive the value and meaning of participating in the community on the other hand, and are more On the other hand, it also makes community members perceive the value and significance of participating in the community, and are more willing to become part of the community and build up their sense of community identity, which in turn leads to brand loyalty. Obviously, community identity plays a mediating role between information experience and brand loyalty.

Similarly, the entertainment experience of consumers in virtual brand community usage contexts can enhance their community identity. Unlike transactional communities where members place the most emphasis on economic value, brand communities emphasize the emotional and psychological satisfaction that comes from social interactions between people (Laroche et al., 2012). Since entertainment itself is one of the most important motivations for consumers to participate in social media, if consumers’ participation in brand communities brings them a sense of relaxation and pleasure and satisfies their hedonistic entertainment experience, it will also significantly enhance the members’ perception of the value and meaning of participating in the community, thus increasing their identification with the community (Cho et al., 2020). Thus, even for functional products that emphasize use value, although their products hardly bring entertainment value directly, the entertainment experience gained from consumers’ participation in their brand community may indirectly influence the relationship between consumers and the brand by forming consumers’ identification with the community (Song, 2015). Therefore, community identity has a mediating role between entertainment experience and brand loyalty.

Information and entertainment experiences enhance community identity among community members primarily by the identity value that comes from participating in the community, whereas interactive experiences enhance community identity primarily through consumer self-categorization and embedding in the community (Haslam et al., 2012). Research suggests that members of a society need to go through a process of Self Categorization (SC), which means that individuals attribute themselves as members of a group and perceive themselves as having certain traits in common with members (Willer, 1989).

In the process of participating in a brand community, community members are constantly exposed to other consumers sharing brand stories and helping other consumers use the brand (Laroche et al., 2012), which invariably strengthens community participants’ shared love for the brand and a common community identity, which provides important conditions for forming a community identity (Song, 2015); at the same time, participation in community interactions is accompanied by the establishment of relationships among community members, and the establishment of community member relationships leads to the creation of reciprocal norms and a sense of shared responsibility in the community similar to those in real interactions (Laroche et al., 2012), which makes community members take the initiative to assume certain roles in the community (e.g., community manager, etc.) to help the community, and this relational community embedding can facilitate consumers to form an identity with the community (Alwash et al., 2021). Therefore, community identity has a mediating role between interactive experience and brand identity. Comprehensive analysis of the above, we thus hypothesize:

**Hypothesis 5.** The community identity mediates the relationship between information experience and brand loyalty.
**Hypothesis 6.** The community identity mediates the relationship between entertainment experience and brand loyalty.
**Hypothesis 7.** The community identity mediates the relationship between interactive experience and brand loyalty.

**FIGURE 1:**
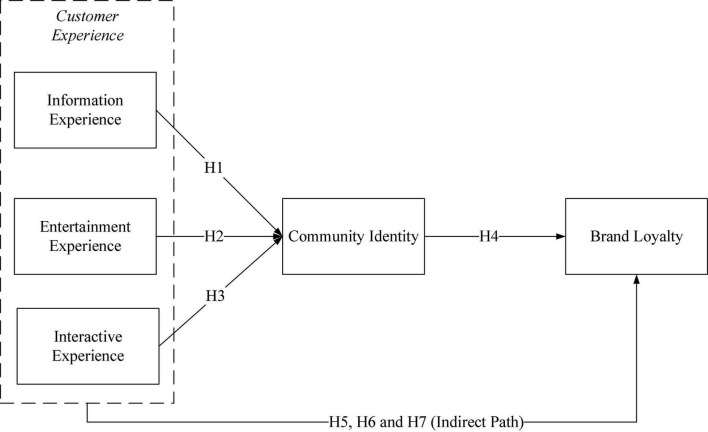
Theoretical model.

In summary, this study proposes a research model as shown in Figure 1.

## Sample and Procedures

### Participants and Procedure

The data collection of this study fully considered the representativeness of the sample, and users of OPPO community were selected as subjects. OPPO community is the official forum of OPPO, which is a virtual platform for OPPO cell phone users to provide cell phone services, resource support and user communication. OPPO community contains detailed cell phone reviews, OPPO cell phone resources, etc. In the OPPO community, users can exchange OPPO cell phones and to the beauty of life without barriers between them, and get the most comprehensive information precipitation; in the OPPO community, not only OPPO customer service online service and solve user problems, but also users can exchange products between them: in the OPPO community, questions and discussions about products, there are senior netizens to answer and interact; in the OPPO community, popular topics discussion section “Entertainment Smorgasbord,” newbie guide section “Newbie Club,” and cell phone photo works exchange section, thus enriching the entertainment exchange topics for OPPO cell phone users and carrying an important function of community friendship. For these reasons, the OPPO community has become one of the most popular virtual brand communities for current consumers, and has a large number of users. Obviously, the user sample of OPPO community is representative. In view of this, this study selects the OPPO community as a virtual brand community as the research object of the questionnaire.

In order to ensure that the respondents were the target audience of this study, two filtering questions were used at the beginning of the survey to determine the eligibility of the subjects, i.e., to exclude users who were under 18 years old and those who were not consistent users. Therefore, consumers who have been consistently using for more than 6 months and are more active were selected as subjects in this study because it is difficult for consumers to have sufficient knowledge about virtual communities if they have been using for a short period of time, and the short period of time also affects consumers’ familiarity with virtual communities, which affects the quality of their responses to the questionnaire. The survey period was from September 18, 2021 to December 16, 2021, and a total of 625 users participated in the questionnaire. 13.92% of the users (*n* = 87) did not answer the trap questions correctly and were therefore excluded from the analysis, and then the questionnaires answered by 560 qualified users were further processed to obtain a valid sample size of 538 after excluding invalid questionnaires.

As shown in the descriptive statistical analysis in Table 1, there are relatively more female users than male users, accounting for 55.95%; in terms of age distribution, the most users are aged 31∼40, accounting for 33.08%, while only 5.57% are aged 50 (inclusive) or above; in terms of marital status, there are far more unmarried users than married users, accounting for a high proportion of 57.63%; in terms of occupation, corporate employees account for the highest proportion, with a proportion of 37.54%; in terms of education, undergraduates account for more, with a proportion of 35.31%; in terms of average monthly consumption, 2000∼5000 yuan or more is the most (59.46%); in terms of continuous use time, 6 months ∼ 1 year (inclusive) users are the most (39.40%), more than 3 years users are the least (25.27%).

**TABLE 1:** Descriptive statistical analysis.

Variables	Item	Frequency	%	Cumulative%
Gender	Male	237	44.05	44.05
	Female	301	55.95	100
Age (year)	20 years old or less	64	11.89	11.89
	21∼30 years old	178	33.08	44.97
	31∼40 years old	182	33.82	78.79
	41∼50 years old	84	15.61	94.40
	51 years old or above	30	5.57	100
Marriage	Married	228	42.37	42.37
	Unmarried	310	57.63	100
Profession	Civil Servants	136	25.27	25.27
	Employees of enterprises	202	37.54	62.81
	Students	64	11.89	74.70
	Other	136	25.27	100
Education level	High school and below	68	12.63	12.63
	College	118	21.93	34.56
	Undergraduate	190	35.31	69.87
	Master’s degree and above	162	30.11	100
Consumption (RMB)	Below 2000 RMB	116	21.56	21.56
	2000∼3999RMB	160	29.73	51.29
	4000∼5999RMB	160	29.73	81.02
	6000 RMB or more	102	18.95	100
Continuous use time (year)	less than 1 year	212	39.40	39.40
	1∼2 years	190	35.31	74.71
	Over 3 years	136	25.27	100

### Measures

In this study, the theoretical model contains a total of five constructs, which are information experience, entertainment experience, interactive experience, community identity, and brand loyalty. In order to ensure the reliability and validity of the measurement scales, the measurement questions in this study were all based on existing established scales, which were compiled through literature analysis according to the purpose of the study. Among them, the scales from previous research were translated and validated according to the recommendations of the back-translation method to ensure the quality of the questionnaire and its applicability. The variables were measured as follows.

(1) Information experience: Three 7-point Likert items was applied to measure this construct. The items measured the degree to which consumers find the information content useful and valuable when they access or share it. the extent to which they participate in the brand’s user community (Sha et al., 2010).
(2) Entertainment experience: The scale items of entertainment experience consist of three items, and the items measured the degree to which consumers participate in a virtual community by gaining a sense of enjoyment and relaxation from participating in that community (Sha et al., 2010).
(3) Interactive experience: The three items measured the degree to which consumers participate in a virtual brand community by interacting and communicating with other members of that community to gain experience (Sha et al., 2010).
(4) Community Identity. The scale items in this study were based on Zheng et al. (2017), and was modified to become a measure of continuance intention based on the actual context of virtual brand community, with contains three items.
(5) Brand loyalty: To measure brand loyalty, we used three items proposed by Delgado-Ballester et al. (2003) and modified into a brand loyalty measurement based on the actual usage context of virtual brand community.

## Data Analyses

### Measurement Model Analysis

In recent years, structural equation modeling (SEM) has become the most popular method of data analysis in social sciences such as management, education and psychology, and is considered a mainstream technique. SEM has become an important tool for testing theories with both experimental and non-experimental data (Jöreskog, 1971). From a statistical methodological perspective, SEM has the unique advantage of handling the data in a meticulous manner, allowing for both the prediction of the remaining estimated residuals and the separation of measurement errors, thus making SEM’s definition of latent variables more consistent with psychometric constructs. Therefore, this study used structural equation modeling software to test and analyze the hypothesized relationships and structural models in the study model. That is, the collected data were analyzed by SEM.

The results of the confirmatory factor analysis are shown in Table 2. This study evaluates Confirmatory Factor Analysis (CFA) measurement model based on a two-stage approach (Anderson and Gerbing, 1998). CFA should report factor loadings of each measurement items, Cronbach’s Alpha, Composite Reliability (CR), and Average Variance Extracted (AVE) for each construct. Table 2 reports the CFA of the measurement models. Among them, factor loadings of all dimensions are between 0.647 and 0.880, Cronbach’s Alpha is between 0.800 and 0.855, and the composite reliability is between 0.800 and 0.855. Convergent Validity is between 0.573 and 0.664, indicating that each construct has adequate convergent validity (Fornell and Lacker, 1981; Hair et al., 2017).

**TABLE 2:** Confirmatory factor analysis.

Construct	Measurement items	Factor loadings	Cronbach’s alpha	Construct reliability (CR)	Average variance extracted (AVE)
Information experience	INFE1: I have access to some useful information or materials in the OPPO community	0.804	0.793	0.800	0.573
	INFE2: I am willing to provide information to other members in the OPPO community	0.809			
	INFE3: When I have a problem, I will go to the OPPO community for information	0.647			
Entertainment experience	ENTE1: I find it interesting in the OPPO community	0.785	0.813	0.814	0.594
	ENTE2: In the OPPO community, I can relax	0.775			
	ENTE3: I can relieve stress in the OPPO community	0.752			
Interactive experience	INTE1: In the OPPO community, I can get support and encouragement from other members	0.650	0.810	0.821	0.608
	INTE2: In the OPPO community, I can communicate with people who have similar ideas	0.880			
	INTE3: In the OPPO community, I can leave a deep impression on other members.	0.792			
Community Identity	COMI1: I am a part of the OPPO community	0.669	0.815	0.811	0.591
	COMI2: I’m happy when someone thinks the OPPO community is good	0.850			
	COMI3: I care about what other members think about the OPPO community	0.776			
Brand loyalty	BRAL1: I will recommend OPPO community to my friends	0.760	0.851	0.855	0.664
	BRAL2: If I change my phone, I will still buy OPPO phone	0.848			
	BRAL3: When buying a cell phone, “OPPO cell phone” is my first choice	0.833			

The results of the discriminant validity analysis are shown in Table 3. Discriminant validity is a measure to test whether any two variables in a theoretical model are identical to each other. This study uses the latest discriminant validity analysis method, namely confidence interval method (Torkzadeh et al., 2003). The confidence interval method is used to confirm the confidence interval of the correlation coefficient between variables. If it fails to include “1,” then it is completely correlated, indicating that the facets have different validity. 95% confidence interval of the correlation coefficient does not involve 1 (see Table 3), which shows the acceptable level of discriminant validity between all the variables.

**TABLE 3:** Discriminant validity.

Parameter	Correlation coefficients	Correlation bias-corrected 95% confidence interval	
		Lower bond (2.5%)	Upper bond (97.5%)
INFE < – > ENTE	0.637	0.523	0.734
INFE < – > INTE	0.635	0.513	0.743
INFE < – > COMI	0.793	0.697	0.862
INFE < – > BRAL	0.708	0.598	0.804
ENTE < – > INTE	0.629	0.504	0.742
ENTE < – > COMI	0.699	0.598	0.782
ENTE < – > BRAL	0.578	0.453	0.702
ENTE < – > COMI	0.733	0.606	0.838
ENTE < – > BRAL	0.746	0.644	0.831
COMI < – > BRAL	0.765	0.644	0.868

*Information experience = INFE; Entertainment experience = ENTE; Interactive experience = INTE; Community identity = COMI; Brand loyalty = BRAL.*

### Structural Model Analysis

The results of the model fit degree analysis are shown in Table 4. The study by Jackson et al. (2009) concluded that in structural models, model fit metrics should be reported as a way to assess, correct, and judge the goodness of measurement models. According to Jackson et al. (2009) criteria, nine goodness-of-fit metrics are usually used to test the model fit. In principle, the lower the χ^2^, the better, but since χ^2^ is sensitive to sample size, the ideal value of χ^2^/df should be less than 5. The results of this study based on Jackson et al. (2009) criteria and applying IBM SPSS AMOS 24 for the analysis are shown in Table 4, all of which met the criteria. Therefore, the structural model of this study has a good model fit.

**TABLE 4:** Model fit criteria and test results.

Model fit	Criteria	Model fit of research model
χ^2^	The small the better	242.273
DF	The large the better	83
Normed Chi-sqr (χ^2^/DF)	< 5	2.919
RMSEA	< 0.08	0.060
SRMR	< 0.08	0.043
TLI	> 0.9	0.953
CFI	> 0.9	0.963
GFI	> 0.9	0.937
AGFI	> 0.9	0.909

### Hypothetical Testing

The regression coefficients are shown in Table 5. Information experience (INFE) (β = 0.353, *p* < 0.001), entertainment experience (ENTE) (β = 0.283, *p* < 0.001) and interactive experience (INTE) (β = 0.165, *p* < 0.001) have a positive and significant impact on community identity (COMI). Therefore, Hypothesis 1, Hypothesis 2 and Hypothesis 3 are supported. Community identity (COMI) (β = 0.922, *p* < 0.001) has a positive and significant impact on brand loyalty (BRAL). Therefore, Hypothesis 4 is supported.

**TABLE 5:** Regression coefficient.

Hypothesis	Unstd. coefficient	S.E.	z-value	Std. coefficient
H1: INFE- > COMI	0.354***	0.045	7.937	0.474
H2: ENFE- > COMI	0.283***	0.042	6.707	0.378
H3: INFTE- > COMI	0.165***	0.049	3.343	0.171
H4: COMI- > BRAL	0.922***	0.071	13.032	0.819

*Information experience = INFE; Entertainment experience = ENTE; Interactive experience = INTE; Community identity = COMI; Brand loyalty = BRAL.*

*** p-value < 0.001.

The results of the intermediary effect analysis are shown in Table 6. In this study, structural equation modeling was used to analyze the mediating effect, and the standard error of the mediating effect was first estimated using Bootstrap estimation technique, and then the significant level of the mediating effect was further calculated. According to Hayes (2009), a mediating effect is indicated if “0” does not include the 95% confidence interval of Bias-corrected, the z-value is greater than 1.96, and the p-value is less than 0.05.

**TABLE 6:** The analysis of indirect effect.

Indirect effect	Path coefficient (β)	SE	Z-Value	p-value	Bootstrap	
					Bias-corrected 95%	
					Lower	Upper
H5: INFE→COMI→BRAL	0.326	0.059	5.525	0.001	0.218	0.453
H6: ENTE→COMI→BRAL	0.261	0.070	3.729	0.003	0.140	0.404
H7: INTE→COMI→BRAL	0.152	0.067	2.269	0.023	0.023	0.293

*Information experience = INFE; Entertainment experience = ENTE; Interactive experience = INTE; Community identity = COMI; Brand loyalty = BRAL.*

Specifically, The indirect effect of information experience on brand loyalty is 0.326, “0” does not include the Bias-corrected 95% confidence interval range, the z-value = 5.525 > 1.96, and the p-value = 0.001 < 0.05. Therefore, there is an indirect effect. The indirect effect of entertainment experience on brand loyalty is 0.261, “0” does not include the Bias-corrected 95% confidence interval range, the z-value = 3.729 > 1.96, and the p-value = 0.003 < 0.05. Therefore, there is an indirect effect. The indirect effect of interactive experience on brand loyalty is 0.152, “0” does not include the Bias-corrected 95% confidence interval range, the z-value 2.269 > 1.96, and the p-value 0.023 < 0.05. Therefore, there is an indirect effect. Therefore, the results of the study showed that hypothesis H5,H6 and H7 was supported.

## Research Results and Discussion

### Research Conclusion

First, community identity plays a partially mediating role between information experience and brand loyalty. The results of this study show that consumers’ information experience in virtual brand communities significantly affects community identity, community identity significantly affects brand loyalty, and community identity plays a partially mediating role. Previous studies have shown that information value is an important motivation that influences consumers’ participation in communities. It is hypothesized that the reason for this may be that when consumers develop some level of knowledge and goodwill toward the brand and are driven by this initial attitude to participate in the virtual brand community to find product information, and when consumers have a good information experience in the community as expected. This not only enhances the consumer’s knowledge of the product, but also drives the consumer to perceive the community members as trustworthy and therefore capable of solving the problems they face when purchasing the product in the future, thus forming an identification with the virtual community, which in turn allows the consumer to form brand loyalty.

Second, community identity plays a partially role between entertainment experience and brand loyalty. The results of this study show that consumers’ entertainment experience in virtual brand communities significantly affects community identity, community identity significantly affects brand loyalty, and community identity plays a complete mediating role. It is hypothesized that the main reason for this is that entertainment is an important motivation for consumers to participate in social media and even communities, and that companies whose consumers participate in virtual communities can use entertaining content to enhance consumers’ favorable perceptions of their brands. This study tested this idea through structural equation modeling and confirmed that consumer entertainment experiences do influence consumer brand loyalty and act through the mediating mechanism of consumer identification with the community.

In addition, community identity plays a partially role between interaction experience and brand loyalty. The results of this study suggest that consumers’ interactive experiences in virtual brand communities significantly affect community identification, and community identification significantly affects brand loyalty; therefore, community identification can mediate the relationship between consumers’ interactive experiences and brand loyalty. It is hypothesized that the reason for this may be that some consumers are motivated to join virtual communities in order to seek the opinions of other consumers, to establish social connections with other consumers, and to form an identity with the community in their interactions with consumers, and this and may influence consumer loyalty to the product.

### Theoretical Contributions

First, we investigated the relationship between community experience and community identity, which enriched the research on the content of community experience. Previous studies have shown that a good community experience can lead consumers to develop positive attitudes toward products and companies, but attitudes do not exactly equal identity (Stoycheff et al., 2017). The research in this paper finds that some community experience dimensions can indeed influence virtual brand community identity, which strongly responds to the value of establishing brand communities for companies.

Second, previous studies have examined the mechanism of community experience as a whole dimension, but the shortcoming is that they cannot find out which dimensions are at work. This study subdivides community experience into three dimensions: information experience, entertainment experience and interactive experience, and examines their effects on brand loyalty separately. The study finds that there are indeed important differences in the influence mechanisms of the three dimensions, which indicates that the influence of community experience cannot be generalized, and companies should manage different experience dimensions in a targeted manner.

Third, this study examines the mechanisms mediating the role of community identity in community experience on brand loyalty. Although, research has considered community identity as one of the important indicators of community performance, academics have not systematically explored the mechanism of community identity generation, this paper reveals the mechanism of community identity generation from the perspective of consumer experience to a certain extent, and enriches the relevant research on community identity in virtual brand communities.

### Practical Implications

First, information experience remains the most important factor influencing consumer brand loyalty. Therefore, it is necessary for companies to communicate timely information about their products or brands, and it is vital to promote knowledge sharing among consumers. From the demand side of product information, consumers’ demand for product knowledge is greatly enhanced due to the increasing complexity of products, and consumers generally trust information released by other consumers more than information released by companies. Therefore, from the supply side of knowledge, enterprises meet consumers’ demand for product information and knowledge not only by relying on product information dissemination initiated by enterprises, but also by promoting knowledge co-creation and sharing among consumers as a virtual brand community, so as to enhance consumers’ information experience and promote the formation of consumers’ loyalty to this brand.

Second, entertainment break has become the most common request of consumers in the social media era. Therefore, enterprises should follow the needs of consumers, change their formal and official information dissemination methods, communicate with consumers in a more personal way, and produce entertaining content to attract consumers, so that consumers can identify with the community and be more willing to buy the company’s products. In the virtual brand community context, consumers’ entertainment experience has become the focus of competition. The relationship between consumers and enterprises is getting closer and closer, and enterprises should transition from “value co-creation” to “experience co-creation” by creating a good entertainment experience to gather consumers and close the distance with them through virtual communities. In short, enterprises can strengthen the connection between enterprises and consumers by establishing virtual brand communities, and strive to create a good entertainment experience for consumers.

Third, while interactive experiences are also a motivation for consumers to participate in virtual brand communities, and a good interactive experience can lead to consumer identification with the community, it is worth noting that interactive experiences are not an end in themselves, but may be a means for consumers to obtain information or for entertainment. The experience economy means that consumers’ focus and attention has shifted from product quality and price to whether they can have a good consumer experience during consumption. Consumers’ pursuit of experience drives brand competition to shift from product and service level to experience level. Since experience can specifically happen in different contexts to produce different forms of experience, such as consumption experience, product experience and service experience. Therefore, companies can create brand experiences for consumers from many elements such as brand design, packaging, and communication. Therefore companies cannot make interaction between consumers an end in itself when they promote it.

### Research Limitations and Future Research Directions

This study uses the OPPO community as a sample to collect data, and the findings are significantly representative, but the sample scope of this study is from mainland China, so the analysis results of the questionnaire data do not reflect relevant research in the field of user community experience in foreign virtual brand communities. Therefore, the theoretical nature of the findings of this study needs to be further verified in a larger scope of different virtual brand communities to further enhance its generalizability in future studies.

In the subsequent research, data collection can be conducted in a broader scope, the community experience impact mechanism of different types of virtual brand communities in the same industry can be verified, empirical analysis can be conducted with a larger sample, as well as a cluster comparison of virtual brand communities in China and developed countries to explore the community experience patterns of consumers in different cultures, regions and countries, so that commonalities and differences can be derived. To provide marketing strategies for virtual brand communities in different market segments and enhance the generalizability of this study’s findings.

## Data Availability Statement

The raw data supporting the conclusions of this article will be made available by the authors, without undue reservation.

## Author Contributions

ZH, YR, and JZ: conceptualization and writing – review and editing. ZH and QW: formal analysis. ZH: investigation. ZH, YR, JZ, QW, and LZ: writing – original draft. All authors have read and agreed to the published version of the manuscript.

## Conflict of Interest

The authors declare that the research was conducted in the absence of any commercial or financial relationships that could be construed as a potential conflict of interest.

## Publisher’s Note

All claims expressed in this article are solely those of the authors and do not necessarily represent those of their affiliated organizations, or those of the publisher, the editors and the reviewers. Any product that may be evaluated in this article, or claim that may be made by its manufacturer, is not guaranteed or endorsed by the publisher.
